# Ethical challenges causing moral distress: nursing home staff’s experiences of working during the COVID-19 pandemic

**DOI:** 10.1080/02813432.2024.2308573

**Published:** 2024-02-09

**Authors:** Annaclara Ariander, Anna Olaison, Christer Andersson, Rune Sjödahl, Lena Nilsson, Lisa Kastbom

**Affiliations:** a Primary Health Care Centre in Johannelund and Department of Health, Medicine and Caring Sciences, Linköping University, Linköping, Sweden; b Department of Culture and Society, Linköping University, Linköping, Sweden; c Department of Orthopedics, Linköping University, Linköping, Sweden; d Department of Surgery and Department of Clinical and Experimental Medicine, Linköping University, Linköping, Sweden; e Department of Anaesthesiology and Intensive Care and Department of Biomedical and Clinical Sciences, Linköping University, Linköping, Sweden; f Department of Health, Medicine and Caring Sciences, Linköping University, Linköping, Sweden; g Primary Health Care Centre in Ekholmen and Department of Health, Medicine and Caring Sciences, Linköping University, Linköping, Sweden

**Keywords:** COVID-19 pandemic, nursing homes, older adults, primary healthcare, qualitative research

## Abstract

**Objective:**

To investigate the experiences of healthcare staff in nursing homes during the COVID-19 pandemic.

**Design:**

Individual interviews. Latent qualitative content analysis.

**Setting:**

Ten nursing homes in Sweden.

**Subjects:**

Physicians, nurses and nurse assistants working in Swedish nursing homes.

**Main outcome measures:**

Participants’ experiences of working in nursing homes during the COVID-19 pandemic.

**Results:**

Four manifest categories were found, namely: *Balancing restrictions and allocation of scarce resources with care needs*; *Prioritizing and acting against moral values in advance care planning*; *Distrust in cooperation* and *Leadership and staff turnover – a factor for moral distress*. The latent theme *Experiences of handling ethical challenges caused by the COVID-19 pandemic* gave a deeper meaning to the categories.

**Conclusion:**

During the pandemic, nursing home staff encountered ethical challenges that caused moral distress. Moral distress stemmed from not being given adequate conditions to perform their work properly, and thus not being able to give the residents adequate care. Another aspect of moral distress originated from feeling forced to act against their moral values when a course of action was considered to cause discomfort or harm to a resident. Alerting employers and policymakers to the harm and inequality experienced by staff and the difficulty in delivering appropriate care is essential. Making proposals for improvements and developing guidelines together with staff to recognize their role and to develop better guidance for good care is vital in order to support and sustain the nursing home workforce.

## KEY POINTS

- The COVID-19 pandemic has affected both patients and staff in nursing homes, in Sweden and worldwide.
- Our study highlights that during the COVID-19 pandemic, nursing home staff encountered several ethical challenges which caused moral distress.
- Moral distress stemmed from not being given adequate conditions to perform their work, thus not giving the residents appropriate care.
- Moral distress could also originate from nursing home staff’s feeling of being forced to act against their moral values.

## Introduction

Since March 2020, the COVID-19 pandemic has posed a threat to societies and healthcare systems worldwide. Before the pandemic, the time between moving into a nursing home and death shortened in Sweden, indicating a population living in nursing homes that is frailer and closer to death [1]. Older, frail people who live in nursing homes have been particularly affected, and have been shown to have an increased mortality rate from COVID-19 in Sweden [2, 3] and worldwide [4–6]. People in nursing homes and those receiving home care services represent 72% of all deaths from COVID-19 among people aged over 70 in Sweden [7].

In Sweden, nursing home care is provided by two authorities in cooperation: regions and municipalities. Nursing homes have attending physicians employed at regional primary healthcare centres (HCCs), usually general practitioners (GPs) or general practitioner specialist trainees. Nurses and other staff are municipality employees. This means that the physicians and nurses and other staff working with nursing home residents have different employers, and also use different patient health record systems. The workforce in nursing homes in Sweden is multiprofessional, with GPs, nurses and nurses assistants who all are jointly responsible for the daily healthcare of patients [8]. Uncertainty around the roles and obligations of staff in nursing homes have been found in previous studies [9, 10].

In the early phase of the pandemic, there was a shortage of personal protective equipment (PPE) in the healthcare system in Sweden [11]. Delivering good care to patients despite the shortage of PPE has been an obstacle during the COVID-19 pandemic [12].

To provide infection control in nursing homes, visiting these units was prohibited from 1 April 2020 until 1 October 2020 throughout Sweden [13]. Due to this restriction, residents in Swedish nursing homes and hospitals more frequently died without the presence of another person; either a loved one or a member of staff. Additionally, the frequency of end-of-life (EOL) discussions was lower during this time compared to deaths in 2019 [14].

During the first wave of the COVID-19 pandemic in Östergötland in Sweden (the region where this study took place), the regional council issued guidelines for advance care planning (ACP) with regard to COVID-19 in nursing homes [15]. These guidelines included standardized phrases that could be used to support facilitating the process of writing care plans in health records. The guidelines included a deadline for documenting ACPs regarding serious respiratory tract infections (i.e. COVID-19) in nursing home residents. The aim of documenting plans, with a focus on serious respiratory tract infections, was to prevent the group from being admitted to hospital if this was unnecessary and/or against the residents’ wishes, and instead to provide these residents with symptom management and good palliative care in their nursing homes.

The healthcare system in Sweden has been subjected to inspections, with several flaws at different levels having been pinpointed. In the inspection of the quality of care in nursing homes during the pandemic carried out by Sweden’s Health and Social Care Inspectorate, several deficiencies were found, for example in connection with staff competence (e.g. medical competence and language skills) and health record documentation, particularly regarding the documentation of EOL discussions with residents [16]. Furthermore, the Coronavirus Commission (i.e. a commission tasked with reviewing the actions of the Government, regions and municipalities to prevent the spread of infection) highlighted the lack of cooperation and coordination between the two authorities providing nursing home care and the different systems for documenting patient health records in these two organizations, among other deficiencies [11].

The impacts of COVID-19 on nursing homes were significant. Therefore, further challenges – both on an organizational level and in the execution of tasks among nursing home staff – are likely to have arisen. Knowledge about the experiences of staff working in nursing homes during the COVID-19 pandemic is still limited. The aim of this study was to investigate the experiences of healthcare staff working in nursing homes during the COVID-19 pandemic.

## Material and methods

### Settings

The ten nursing homes included in the present study were long-term care homes, including care homes for patients with dementia. Units for short-term care only (care homes for patients waiting to be transferred to long-term care homes or to ordinary homes after being discharged from hospital) were not included. Participants were recruited from ten nursing homes, and the physicians were employed at seven different HCCs. Both rural and urban nursing homes were represented in several municipalities of the region studied (see Table 1). The manager of each unit was contacted by e-mail with information about the study and the manager subsequently reported back to the research group potential participants who had agreed to be contacted for further information about the study.

**Table 2.** Examples of steps of the analysis using qualitative content analysis.

Meaning unit	Code	Manifest category	Latent theme
‘She faded away a little bit faster than she would have done if there had been more social contact.’	Faded away faster. Social isolation. Visitation restrictions.	Balancing restrictions and allocation of scarce resources with care needs	Experiences of handling ethical challenges caused by the COVID-19 pandemic
‘We had to do it, because the scenario was that many will get ill and who should we prioritize? So actually, the decision was not that hard for me.’	Had to make care plans. Scenario of many infected. Prioritizing healthcare. Moral values.	Prioritizing and acting against moral values in advance care planning	Experiences of handling ethical challenges caused by the COVID-19 pandemic
‘… I felt that society prioritized the region [i.e. not municipal units] with personal protective equipment and so on. And forcefully proclaimed that the staff were strained. While the municipalities’ preparedness sadly… The intent was there, but there was no equipment, there was no possibility to protect ourselves.’	Prioritizing the region units, not the municipal ones. A need to be better prepared for serious incidents. Measures could have been taken to better protect nursing home units. Leadership deficiency.	Leadership and staff turnover – a factor for moral distress	Experiences of handling ethical challenges caused by the COVID-19 pandemic
‘People with multiple comorbidities, who have decreased autonomy, who are frail, they are in the hands of the caregivers. And what care they get is dependent on what we know about them.’	Nursing home residents in the care givers’ hands. Decreased autonomy. Dependent on others. Staff continuity associated with properly functioning nursing home.	Distrust in cooperation	Experiences of handling ethical challenges caused by the COVID-19 pandemic

### Participants and interviews

The inclusion criteria were as follows: Working as healthcare staff at a nursing home, speaking Swedish and agreeing for the interview to be recorded digitally. Participants were recruited through purposeful sampling [17] from nursing home units which had been affected the most and the least by COVID-19-related deaths [18].

An interview guide was developed by the research group with open questions about the participants’ experiences, such as: ‘Please tell me about your experiences from working at a nursing home during the COVID-19 pandemic’, ‘What were the greatest challenges regarding testing nursing home residents for COVID-19?’ and ‘How were information and guidelines communicated to the physicians/nurses/other staff at the nursing home?’. Clarifying questions were asked [19]. These follow-on questions picked up on ambiguities, and aimed to understand how these uncertainties came to make sense within the participants’ stories.

A physician who is also a GP (the last author of this paper; LK) performed the 20 interviews (between February and April 2022), which were recorded digitally and transcribed verbatim. The length of each interview varied between 30 and 70 min. The study was approved by the Swedish Ethical Review Authority (Dnr. 2021-06691-01).

### Analysis

The interviews were analysed using latent qualitative content analysis. This method allows for an open-ended analysis with no preconceived categories, allowing the categories to emerge from the data [20]. The analysis was performed using the following seven steps: (1) The transcribed interview was read through to obtain an overall impression and to get a broad understanding. (2) Segments of the texts dealing with the aim of the study were identified, and meaning units were constructed through a close reading of the text. (3) The meaning units were condensed and abstracted into codes. (4) The codes were compared and sorted into categories. (5) The categories were compared to the entire interview, to make sure that the interpretation was consistent and coherent with the text as a whole. These categories were compared to avoid overlapping, and content descriptions were developed. (6) The latent content of the text emerged through an interpretation of the underlying meaning. As a result of this process one latent theme emerged as a deeper meaning of all the manifest categories in the analysis. (7) Quotations were used to exemplify the categories [20]. Examples of the steps of the analysis are shown in Table 2.

The preliminary categories were mainly coded by the first and last authors (AA and LK). Validity was built into the analysis by testing tentative categories until saturation was reached by the entire research group. As part of the reflexivity process, the categories were endorsed by complementing and challenging each other’s readings and preunderstandings [19].

## Results

**Figure 1.**
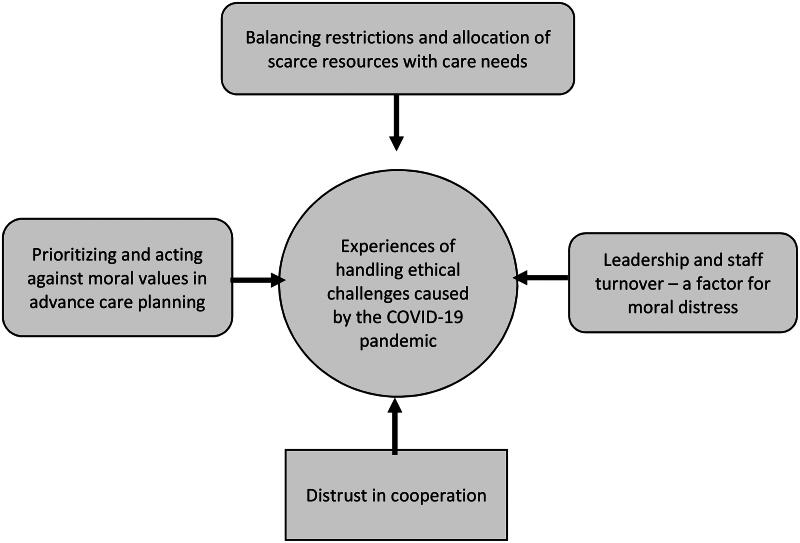
Overview of manifest categories and theme describing experiences of working in nursing homes during the COVID-19 pandemic.

An overview of the participants is presented in Table 1. When analysing the data using qualitative content analysis [20], four manifest categories were identified. One latent theme, *Experiences of handling ethical challenges caused by the COVID-19 pandemic*, emerged to give a deeper meaning to the categories (See Figure 1).

**Table 1.** Characteristics of participants.

Sex, men/women (n(%))	6 (30)/14 (70)
Age, mean (range)	49 years (31–72)
Years worked since degree, mean (range)	21 (2–45)
Years worked at nursing homes, mean (range)	15 (0.5–40)
Physicians (n(%))	11 (55)
Nurses (n(%))	4 (20)
Nurse assistants (n(%))	5 (25)
Primary healthcare centres* (n)	7
*Primary healthcare centres, rural (n(%))*	*3 (43)*
*Primary healthcare centres, urban (n(%))*	*4 (57)*
Nursing homes** (n)	10
*Nursing homes, rural (n(%))*	*5 (50)*
*Nursing homes, urban (n(%))*	*5 (50)*

* Employers for the participants being physicians.

** Employers for the participants being nurses and nurse assistants.

### Balancing restrictions and allocation of scarce resources with care needs

The use of PPE when caring for the residents was perceived as a necessity. The lack of such equipment in nursing homes during the early stages of the pandemic caused insecurity and fear among the staff. Wearing protective equipment was complex. Whilst the fear of being contracting COVID-19 was reduced by using PPE, participants also found the equipment as uncomfortable, for example by causing a feeling of not being able to breathe properly by causing mist on the visors making it difficult to see properly. The equipment was also described as a barrier to communication, particularly in cases where the resident’s hearing was impaired. Wearing the protective equipment could also make staff feel that residents did not recognize them, particularly when caring for residents with cognitive impairments.

The testing capacity during the later stages of the pandemic was adequate, and there was a low threshold for testing for COVID-19. Some thought that the excessive spread of infection among residents in some of the units was caused by the fact that testing for COVID-19 was limited and not easily available in nursing homes during the earlier stages of the pandemic. Testing the residents was ethically problematic when the residents were reluctant to be tested, especially when they did not comprehend what was happening, e.g. residents with cognitive impairments. For some, testing residents who were reluctant to be tested felt like an assault, which sometimes posed an ethical challenge, putting the two ethical principles autonomy and beneficence in conflict. The use of coercion when testing was perceived by some as necessary to prevent the spread of infection.

Isolation of a resident due to symptoms of COVID-19 was described as challenging, especially when the resident was cognitively impaired. Therefore, it was not always possible to adhere to current infection control guidelines.

Visitation restrictions impacted the residents negatively, according to the participants, who described how residents seemed worried and depressed to a larger extent than usual during this period. The visitation restrictions also impacted the residents’ family members, who were found to be more anxious and needed information about the residents’ physical status to a greater extent than prior to the restrictions. Sometimes, the participants thought that the social isolation could negatively impact the quality of the residents’ lives, and even accelerate the dying process.

She faded away a little bit faster than she would have done if there had been more social contact. [P20. Woman. 37 years old. Physician.]

The visitation restrictions were described by some as necessary to limit the spread of infection. Others believed that it was ethically indefensible to deny the residents their right to see their loved ones, that it was a violation of their autonomy, putting the principles of beneficence non-maleficence in conflict.

What is life worth then, what are we saving them from when they cannot even see their loved ones? [P17. Woman. 62 years old. Physician.]

There were times at the beginning of the pandemic when the visitation restrictions prohibited loved ones from coming into close contact with the residents at the end of their lives. This was perceived as agonizing.

Those who became ill and died have had to do so without the opportunity to have contact with their loved ones. [P18. Man. 72 years old. Physician.]

However, others expressed that when a resident was terminally ill and death was imminent, an exception was made and visits were allowed with some restrictions, e.g. visitors in the resident’s home only.

The lack of physical touch contributed to the residents experiencing poor psychological health. Several participants also described the act of watching over a dying person as undignified due to the use of PPE.

One woman was sitting with her mother who was really ill. She sat fully clothed in personal protective equipment. […] And to touch your mother, I mean, this woman has given birth to me, she has cared for me during my whole upbringing and supported me during adulthood, and here I am touching my mother wearing gloves. [P4. Woman. 48 years old. Nurse assistant.]

### Prioritizing and acting against moral values in advance care planning

Physicians participating in the study found that creating and updating ACPs with regard to COVID-19 for all nursing home residents could not be done using the standard method for ACP (i.e. visiting the patient for a discussion with the patient and/or family members regarding the content and direction of care) within the timeframe that was given. Furthermore, the visitation restrictions meant that physicians refrained from visiting the nursing homes. Some physicians used the standardized phrases that were suggested and documented a care plan without prior discussions with the resident and/or family members. Some, often those who had prior knowledge about the resident, found that this method was adequate for a care plan.

Some thought that the process of creating ACPs was helpful in the care of the nursing home residents, and that it was necessary given the situation of the spread of infection within society. Others experienced that the COVID-19-specific ACPs were never used in clinical work, and that subsequent rewriting of the care plans was needed.

We had to do it, because the scenario was that many will get ill and who should we prioritize? So actually, the decision was not that hard for me. [P17. Woman. 62 years old. Physician.]

The ACP guidelines were perceived as mandatory rather than as suggestions, which caused moral distress for the physicians who were responsible for the ACP production and sometimes made them feel that they were forced to act against their moral values. The moral distress stemmed from feeling forced to produce ACPs hastily, and as a result, of poor quality, not meeting the physicians’ standard.

### Leadership and staff turnover – a factor for moral distress

Regional guidelines were updated frequently, which resulted in difficulties staying up to date. The need to alter the guidelines was described as a necessity in order to deal with the spread of infection. However, the guidelines were perceived as vague, which caused confusion about how to act in different situations. Some also pointed out that the written guidelines were not adapted to nursing homes, and did not take into account the difficulties involved in providing optimal care regarding infection control in nursing homes specializing in dementia care.

Cooperation between the municipality and the region in the nursing home care was sometimes poor. Some barriers were identified, such as the difficulty of having two different systems for documenting patient health records. Some found it difficult to apply infection control measures in the nursing home context if a resident showed symptoms of COVID-19. This was sometimes described as an example of the difficulties of having different objectives, i.e. the municipality focusing on nursing care and the region focusing on medical care. In addition, the different authorities providing care in nursing homes gave different information to their staff, which led to confusion among the staff. A delay in informing the municipalities was described.

Some proposed that the leadership of the municipalities and regions should have been united, and that medical staff should have been involved in establishing guidelines to improve the content and make implementation easier in nursing homes. In some nursing homes, the guidelines were revised by medical staff at the unit, which was perceived as positive.

The leadership varied between different nursing homes. Some experienced leadership as being present, while some experienced it as absent. An absent manager caused more uncertainty among the staff. Some felt abandoned by the managers, with a lack of appreciation. In cases where nursing home staff felt more appreciated by their managers, staff were more likely to thrive at work during the pandemic.

Participants described a need to be better prepared for serious incidents. The pandemic caught nursing homes off guard, and measures could have been taken to prevent the PPE shortages and to prepare guidelines to ensure nursing homes were better protected against the spread of infection.

We did not understand what this would mean to us. […] I felt that society prioritized the region [i.e. not municipal units] with personal protective equipment and so on. And forcefully proclaimed that the staff were strained. While the municipalities’ preparedness sadly… The intent was there, but there was no equipment, there was no possibility to protect ourselves. [P3. Man. 57 years old. Nurse.]

### Distrust in cooperation

Staff continuity was referred to as a factor associated with a properly functioning nursing home, and as a condition for delivering good quality care to the residents.

People with multiple comorbidities, who have decreased autonomy, who are frail, they are in the hands of the caregivers. And what care they get is dependent on what we know about them. What we know about their diseases. [P18. Man. 72 years old. Physician.]

A factor that was viewed as promoting the spread of COVID-19 was staff discontinuity and turnover. Although having several temporary workers was considered a risk for spreading the infection, this was also described as necessary to ensure sufficient care for the nursing home residents, e.g. when regular staff were absent due to sick leave when displaying infection symptoms. In addition, staff shortages were attributed to poor working conditions and heavy workloads. The distorted dynamics experienced by staff due to a lack of personal continuity, i.e. not knowing who was on duty, which meant that they could not work as a close-knit team around the residents. The combination of these factors created a distrust in cooperation for the staff.

Offering staff training and occupation specific education was presented by the participants as a strategy for improving the care offered to the residents, as well as promoting employee satisfaction and preventing staff turnover.

### Experiences of handling ethical challenges caused by the COVID-19 pandemic

The latent theme *Experiences of handling ethical challenges caused by the COVID-19 pandemic* emerged to give a deeper meaning to all the manifest categories. The demand to produce ACPs caused moral distress for the physicians who created and documented them. The moral distress regarding ACPs could have stemmed from not being given satisfactory conditions to perform their tasks correctly, and thus not being able to give the residents adequate care. Moreover, the feeling of not having a choice regarding whether or not to write the care plans caused moral distress. The participating physicians perceived the information about the ACPs from their managers to be a mandatory task that they could not refuse to carry out. Why it was perceived as a mandatory task is unclear, but could be explained by the way this was communicated to physicians, as no repercussions were presented when failing to produce the ACPs in the given timeframe. The fact that physicians felt forced to perform tasks which they felt were wrong caused moral distress.

For some, the visitation restrictions – and sometimes even testing residents against their will – posed moral distress, and gave rise to questions like: ‘To what extent is it acceptable to cause harm to one person in order to protect another?’ For some nursing home residents, the consequences of the visitation prohibition could be sadness, loneliness and sometimes even dying alone. Testing for COVID-19 could mean putting a resident in an unpleasant situation, since they might perceive testing as frightening if they did not understand what was happening and the reason for it. The ethical challenge aroused from balancing doing harm to one resident (i.e. principle of non-maleficence), whilst protecting other residents from the infection (i.e. principle of beneficence).

Some felt that restricting the care provided to residents, by writing ACPs stating that the resident should not be sent to hospital when displaying symptoms of a serious lower respiratory tract infection (i.e. COVID-19), diminished their value.

What is a human worth when she suffers from a cognitive impairment? Is she not a human? Who decides? [P12. Woman. 41 years old. Nurse.]

When dealing with these different ethical challenges during the COVID-19 pandemic, participants obtained support from colleagues. This was described as the one factor that relieved the moral distress experienced when working in a nursing home during the pandemic.

## Discussion

### Statement of principal findings

This study highlights that during the COVID-19 pandemic, staff working in nursing homes encountered several ethical challenges which caused moral distress. The moral distress stemmed from not being given adequate conditions to perform their work properly, and thus not being able to give the nursing home residents adequate or appropriate care. Another aspect of moral distress originated from staff’s feeling of being forced to act against their moral values when a course of action in the nursing homes was considered to cause discomfort or harm to a resident.

### Strengths and weaknesses of the study

The selection of healthcare workers in nursing homes is broad, but each category of personnel is small (physicians: n11, nurses: n4, nurse assistants: n5), which could be seen as a weakness of the study. However, we find a variation regarding views in each question studied, and find the presentation of different occupations to be positive. Data from both physicians, nurses and nurse assistants were present in all the manifest categories. However, a qualitative comparison between the occupations were not made in the analysis.

One of the strengths of the present study is that it included the experiences of a broad range of nursing home staff regarding occupation, sex, age and numbers of years in the profession. Participants were recruited from nursing homes in rural and urban locations in the region studied, and both long-term homes and units for patients with dementia were included. The nursing homes selected represented both units that were severely affected by the outbreak of COVID-19 and units that were spared from serious spread of infection. The broad variety of participants supports the generalizability of the findings, and the results are transferable to similar settings in nursing homes. Finally, the involvement of researchers with experience in qualitative research, and from various scientific disciplines, such as primary care, surgery, orthopaedics, anaesthesia, patient safety and social work, provided an opportunity to validate the findings through analyst triangulation [17, 20]. The interviewer was a GP, with previous experience of interviewing nursing home staff and with clinical experience from working in nursing homes.

### Findings in relation to other studies

In the present study, we describe the experiences of healthcare staff working in nursing homes during the COVID-19 pandemic. Several obstacles to providing good quality care to the residents were identified, causing a number of aspects of ethical challenges for the participants.

#### Experiences of handling ethical challenges caused by the COVID-19 pandemic

Medical ethics can be described using four ethical principles, namely: respect for autonomy, beneficence, non-maleficence and justice [21]. The status of ethical principles is best described as ‘prima facie’, meaning that a principle is a duty that is binding on all occasions unless it conflicts with equal or stronger duties [22]. Adherence to principles can be difficult because they can conflict, so it can be critical to determine the overriding duty by finding the ‘greatest balance’ of right and wrong in each case where there is a conflict between principles. Having to choose between the ethics of professional duties and one’s personal values can create morally distressing situations for staff. Moral distress is a prominent phenomenon in the health professions and has been studied previously in the healthcare field [23, 24]. In this study, we base our understanding of the concept of moral distress on Jameton [25], who introduced it to the healthcare literature and defined it as ‘when one knows the right thing to do but institutional constraints make it nearly impossible to pursue the right course of action’ (p. 6).

In our study, it was clear that the pandemic was causing conflicts between ethical principles and that this gave rise to moral distress in different ways.

#### Balancing restrictions and allocation of scarce resources with care needs

The aim of enforcing the visitation restrictions in nursing homes was to protect the residents from the spread of COVID-19 and was thereby an act of beneficence. However, enforcing visitation restrictions also resulted in residents becoming isolated and, for some residents, causing poor psychological health, which resulted in doing harm to them. The visitation restrictions also violated the residents’ rights to autonomy and therefore posed an ethical challenge causing staff to experience moral distress, even though the decision was not made by them. The perception of moral distress caused by this ethical challenge may have stemmed from having to enforce the visitation restrictions and seeing at first hand the consequences of the guidelines.

The high prevalence of frailty in nursing homes [26] probably impacted how the visitation restrictions were perceived. This can, in part, be explained by the changed direction of care during the later stages of life [13, 26]. For some, the visitation restrictions significantly impacted nursing home residents’ quality of life [27, 28]. If the aim was to regain quality of life, this could be seen as an act of non-beneficence [21, 22].

When coercion was used in nursing homes during the COVID-19 pandemic, the participants in the present study found this to be morally stressful. This can be partly explained by two conflicting values, namely beneficence and non-maleficence. The moral distress could have been enhanced by the fact that the aim of achieving beneficence was not always directed towards the resident being affected, but towards other residents in the nursing home. Similarly, McLean et al. [29] found that physicians experienced ethical challenges, due to the conflicts involved in providing good care to one resident while trying to protect others.

#### Prioritizing and acting against moral values in advance care planning

Establishing care plans during the COVID-19 pandemic was a source of moral distress. The process of producing ACPs involves balancing beneficence, non-maleficence and autonomy [8]. It is therefore not surprising that this process can cause moral distress. Further, the participating physicians in this study described a feeling of not being able to perform their jobs in a proper manner when establishing the ACPs during the pandemic. One of the components of the ACPs during the COVID-19 pandemic was deciding whether or not the resident should receive hospital care in the event of contracting a COVID-19 infection. This type of decision is usually made carefully through discussions with the resident and/or family members. The request to establish ACPs within a tight timeframe might have contributed to uncertainty about whether or not the decision was of beneficence for the resident, thus generating moral distress.

Decisions on treatment limitations – such as hospital care limitations, i.e. the patient should never receive hospital care, or hospital care only in the case of certain situations – have also been made prior to the pandemic. However, the participants described a feeling of diminishing a resident’s value with regard to the right to receive care as well as being involved in decisions concerning treatment and care when care was restricted during the COVID-19 pandemic. This is in line with results from McLean et al. [29], who found that physicians in Norway working in nursing homes during the COVID-19 pandemic experienced difficulties in prioritizing care. The emphasized moral distress perceived during the pandemic could in part be attributed to the shortage of hospital beds, which might have impacted the medical decisions made in nursing homes, thus resulting in another aspect of restricting care.

One way of dealing with the perceived moral distress was talking to co-workers about the issues and thereby supporting each other. When studying the prevalence of ethical issues in nursing homes in Norway, Bollig et al. found that ethical challenges often arouse regarding EOL discussions, coercion and lack of recourses, and that meetings to discuss these issues could help resolve the issues and relieve the moral distress [30]. Clear guidelines to improve the nursing assistants’ work environment and a need for an outlet where they can reflect on their working environment and their experiences were described as important in a Swedish nursing home context during the first wave of the COVID-19 pandemic by Bergqvist et al. [31].

#### Leadership and staff turnover – a factor for moral distress

Leadership was described as varied, some feeling supportive and present managers, while others experienced an absence of leadership, sometimes leading to a feeling of abandonment. This is in line with Rücker et al. who concluded that avoiding confusion about PPE and having present leadership could be factors for improving strategies for dealing with another pandemic [32]. White et al. [33] even suggested that the working conditions of nursing home staff during the COVID-19 pandemic were a factor that could lead to increased burn-out, staff turnover, and staff shortages. This was similar to the findings of the present study, as the participants highlighted problems with discontinuity due to staff turnover during the pandemic, which led to unsatisfactory working conditions. According to the participants in this study, one aspect that was described as problematic during the pandemic was the frequent changes and revisions of guidelines, which led to confusion and uncertainty in day-to-day work.

### Conclusions and clinical implications

The present study implies that the working conditions during the COVID-19 pandemic impacted nursing home staff in several ways. Facing different aspects of ethical challenges caused moral distress to the staff caring for nursing home residents. Moral distress could arise from not being able to provide care that lived up to the individual’s standards, due to both factors such as frequently altered and revised guidelines, applying visitation restrictions and testing residents for COVID-19, and also due to staff sometimes viewing these guidelines as barriers for good nursing care. The results of this study have implications for healthcare staff, particularly staff in a nursing home context. The study shows that there are opportunities at every level to make a difference and to support nursing home staff. It is essential that employers and policymakers are made aware of the potential harm to residents, the inequalities in the working conditions of staff during the pandemic and the difficulties in providing appropriate care. Further, making proposals for improvements and developing guidelines together with nursing home staff about how to operate safely and ethically, recognizing the role of staff and developing better guidance for maintaining good care are seen as strategies for success.
